# Developing a screening instrument for predicting psychological morbidity after critical illness

**DOI:** 10.1186/cc12416

**Published:** 2013-03-19

**Authors:** A Schandl, M Bottai, E Hellgren, Ö Sundin, P Sackey

**Affiliations:** 1 Karolinska University Hospital Solna, Stockholm, Sweden; 2 Karolinska Institutet, Stockholm, Sweden; 3 Mid Sweden University, Östersund, Sweden

## Introduction

The purpose was to develop a predictive screening instrument for use at ICU discharge to identify patients at risk for post-traumatic stress, anxiety or depression.

## Methods

Potential risk factors for psychological problems were prospectively collected at ICU discharge. Two months after ICU discharge 252 ICU survivors received the questionnaires Post-Traumatic Stress Symptom scale-10 (PTSS-10) and Hospital Anxiety and Depression Scale (HADS) to estimate the degree of post-traumatic stress, anxiety and depression.

## Results

Of the 150 responders, 31% had adverse psychological outcome, defined as PTSS-10 >35 and/or HADS subscales ≥8. After analysis, six predictors with weighted risk scores were included in the screening instrument: major pre-existing disease, being a parent to children younger than 18 years of age, previous psychological problems, in-ICU agitation, being unemployed or sick-listed at ICU admission and appearing depressed in the ICU. Each predictor corresponded to a given risk score. The total risk score, the sum of individual risk scores, was related to the probability for adverse psychological outcome in the individual patient. The predictive accuracy of the screening instrument, as assessed with area under the receiver operating curve, was 0.77. When categorizing patients in three risk probability groups - low (0 to 29%), moderate (30 to 59%) and high (60 to 100%) risk - the actual prevalence of adverse psychological outcome in respective groups was 12%, 50% and 63%.

## Conclusion

The preliminary screening instrument may aid ICU clinicians in identifying patients at risk for adverse psychological outcome after critical illness. Prior to wider clinical use, external validation is needed.

